# *Equid Alphaherpesvirus 1* (EHV-1) Influences Morphology and Function of Neuronal Mitochondria In Vitro

**DOI:** 10.3390/pathogens11080876

**Published:** 2022-08-03

**Authors:** Marcin Chodkowski, Anna Słońska, Karolina Gregorczyk-Zboroch, Zuzanna Nowak-Zyczynska, Anna Golke, Małgorzata Krzyżowska, Marcin W. Bańbura, Joanna Cymerys

**Affiliations:** 1 Division of Microbiology, Department of Preclinical Sciences, Institute of Veterinary Medicine, Warsaw University of Life Sciences, 02-786 Warsaw, Poland; anna_slonska@sggw.edu.pl (A.S.); anna_golke@sggw.edu.pl (A.G.); marcin_banbura@sggw.edu.pl (M.W.B.); 2 Military Institute of Hygiene and Epidemiology, Kozielska 4, 01-163 Warsaw, Poland; malgorzata.krzyzowska@wihe.pl; 3 Division of Immunology, Department of Preclinical Sciences, Institute of Veterinary Medicine, Warsaw University of Life Sciences, 02-786 Warsaw, Poland; karolina_gregorczyk_zboroch@sggw.edu.pl; 4 Department of Animal Genetics and Conservation, Faculty of Animal Breeding, Bioengineering and Conservation, Warsaw University of Life Sciences-SGGW, 02-786 Warsaw, Poland; zuzanna_nowak@sggw.edu.pl

**Keywords:** EHV-1, neuronal cell culture, mitochondria, mitophagy, ROS

## Abstract

Mitochondria are key cellular organelles responsible for many essential functions, including ATP production, ion homeostasis and apoptosis induction. Recent studies indicate their significant role during viral infection. In the present study, we examined the effects of equine herpesvirus type 1 (EHV-1) infection on the morphology and mitochondrial function in primary murine neurons in vitro. We used three EHV-1 strains: two non-neuropathogenic (Jan-E and Rac-H) and one neuropathogenic (EHV-1 26). The organization of the mitochondrial network during EHV-1 infection was assessed by immunofluorescence. To access mitochondrial function, we analyzed reactive oxygen species (ROS) production, mitophagy, mitochondrial inner-membrane potential, mitochondrial mass, and mitochondrial genes’ expression. Changes in mitochondria morphology during infection suggested importance of their perinuclear localization for EHV-1 replication. Despite these changes, mitochondrial functions were preserved. For all tested EHV-1 strains, the similarities in the increased fold expression were detected only for *COX18, Sod2*, and *Tspo*. For non-neuropathogenic strains (Jan-E and Rac-H), we detected mainly changes in the expression of genes related to mitochondrial morphology and transport. The results indicate that mitochondria play an important role during EHV-1 replication in cultured neurons and undergo specific morphological and functional modifications.

## 1. Introduction

Mitochondria are the essential cellular organelles responsible for many functions, the most important of which is ATP production during oxidative phosphorylation [1]. Other tasks of mitochondria include maintaining normal Ca^2+^ ion homeostasis, lipid peroxidation, free radical scavenging, production of NADH and GTP in the citric acid cycle, and synthesis of phospholipids for cell membrane biogenesis. Mitochondria also act as specific signaling centers of the cell, for example, by receiving signals leading to apoptosis [2,3]. 

The shape, size, number, and distribution of mitochondria are closely related to cell function and metabolic activity. Nerve cells, muscle cells, or liver cells have many mitochondria compared to erythrocytes, which have lost them [4]. Mitochondria are distributed evenly throughout the cytoplasm or located peripherally. They are very dynamic and can move to different cell regions depending on the energy requirements. Most often, they are located in the perinuclear space, which is directly involved in protein biosynthesis [5].

The main functions of mitochondria in the cell are well-known. However, their role during viral infection is not fully understood. The most common viral strategy described in the literature is “hijacking” the mitochondria, which allows the virus to replicate efficiently and to take control over the entire cell metabolism [6]. Viruses alter the functions, morphology, and distribution of the mitochondrial network in infected cells. For example, the HBV core protein localizes to the OMM, disrupting electron transport and increasing ROS. Other studies indicate increased mitochondrial network fission dependent on Drp1 protein during HCV infection [7]. In addition, the HCV viral protein NS5A, interacting with phosphatidylinositol 4-kinase IIIα to cause mitochondrial fragmentation, has also been identified [8]. For Dengue virus, two proteins causing increased mitochondrial fission have also been identified: the NS4B and NS3 DENV proteins. Influenza virus H1N1, on the other hand, classified as an RNA virus, causes mitochondrial elongation, into tubular forms, by increasing Opa1 protein expression [9]. The well-known recent SARS-CoV-2- interacts with mitochondrial transport proteins, e.g., TOM70, which may also translate into mitochondrial morphology by affecting the MAVS protein [10]. In addition, the study by Chodkowski et al. indicates increased mitochondrial network fragmentation in human keratinocytes following HHV-1 infection [6]. Many types of viral proteins have been identified as responsible for the modulation of apoptosis. Alphaherpesviruses, like other large DNA viruses, encode proteins that interfere with mitochondrial function and localization to block the apoptotic pathway. Some studies suggest that viruses can eliminate mtDNA, taking complete control over ATP production for “their own needs”. An example is the human herpesvirus type 1 (HHV-1). The HHV-1 UL12.5 protein exhibits nuclease activity, thereby degrading mtDNA [11]. 

EHV-1 belongs to the family of herpesviruses and the subfamily of alphaherpesviruses like the widespread HHV-1. It poses a significant risk to horses due to its pathogenicity. The clinical signs associated with infection may be mild, such as respiratory tract inflammation. On the other hand, it can cause abortions, fetal death and severe neurological complications. Like HHV-1, it shows the ability to establish a latency state. Therefore, studies using EHV-1 provide a good model for research on alphaherpesviruses. Equid alphaherpesviruses show tropism to nerve cells, but information on the pathogenesis of EHV-1 infection, with particular emphasis on virus–neuron cell interactions, is still lacking. So far, the involvement of individual cytoskeleton elements during EHV-1 infection and modulation of apoptosis or autophagy have been determined [12,13,14,15]. The following work is a continuation of research into the role of cellular elements in EHV-1 infection. The aim of this study was to investigate the effects of EHV-1 infection on mitochondrial function in neurons. 

## 2. Results

### 2.1. Mitochondrial Morphology

**Figure 1:**
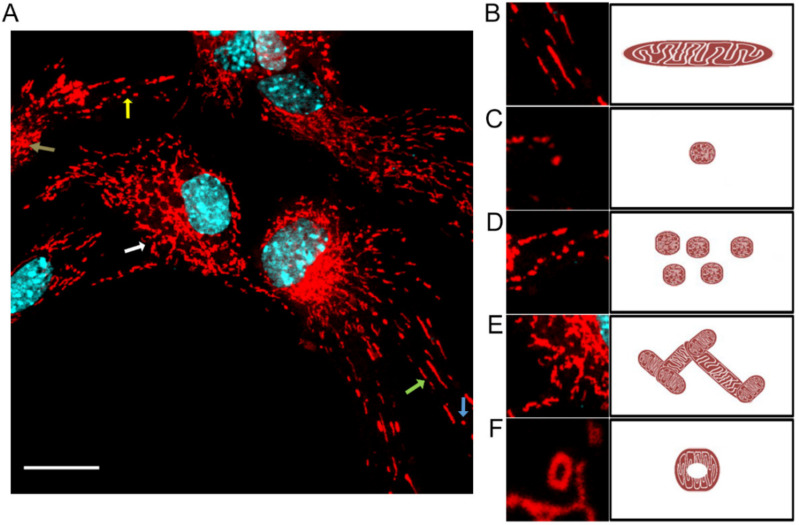
Uninfected neurons demonstrate many tubular, long, and highly interconnected mitochondria (**A**). White arrows indicate branched mitochondria (**E**); yellow—group punctate (**D**), green—long, tubular (**B**); blue indicate single punctate of mitochondria (**C**), brown arrow indicate donut-like mitochondria (**F**). Mitochondria—red fluorescence, nuclei—blue fluorescence. Scale bar 20 µm.

Different morphological types of mitochondria were identified in primary murine neurons (Figure 1). The dominant form was tubular mitochondria, characterized by an elongated shape, arising from the fusion of punctate mitochondria (Figure 1B). We could find single punctate mitochondria (Figure 1C), and also in groups (Figure 1D). In addition, mitochondria forming a highly elaborate network with numerous branches (Figure 1E) were observed. Immunofluorescence images also identified donut-type mitochondria (Figure 1F).

**Figure 2:**
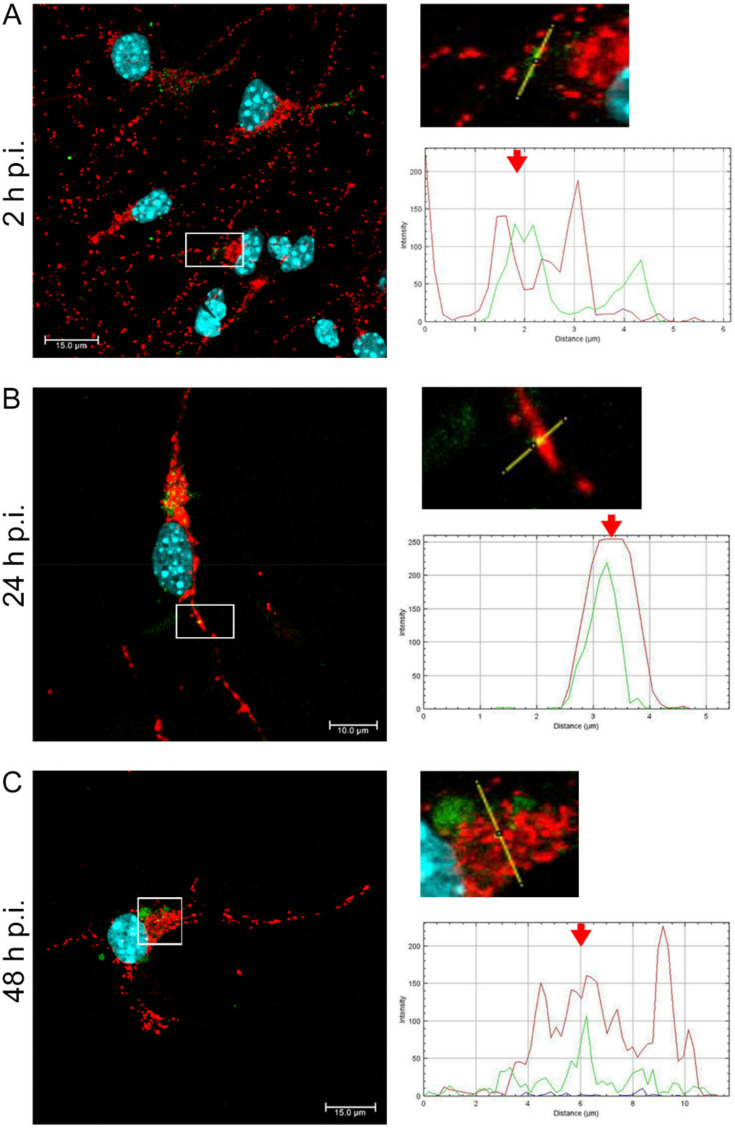
Jan-E EHV-1 infection results in disorganized mitochondria without inter-connections, while the branched network is relaxed. Mitochondrial network morphology in neurons during Jan-E EHV-1 infection (**A**–**C**) at three time points (2, 24, and 48 h p.i., respectively). The line profile plots indicate the intensity distribution of green (viral antigens) and red (mitochondria) channels. Colocalization analysis was performed along yellow lines in the magnified view of ROI in the merged panel using ImageJ software. Red arrows show the overlay of the red and green channels indicating colocalization (mitochondria—red fluorescence, viral antigens—green fluorescence, nuclei—blue fluorescence).

After infection with the Jan-E EHV-1 (Figure 2), we observed enhanced mitochondrial network fission at 2, 24, and 48 h post infection (h p.i.) (Figure 2A). Furthermore, we detected a complete colocalization of viral antigens with mitochondria at 24 h p.i. (Figure 2B), and partial colocalization at 2 and 48 h p.i. (Figure 2A,C). Accumulation of viral antigens in the perinuclear space was found at 2, 24, and 48 h p.i., while at 48 h p.i., EHV-1 was detected in the peripheral parts of the neurons (Figure 2). 

**Figure 3:**
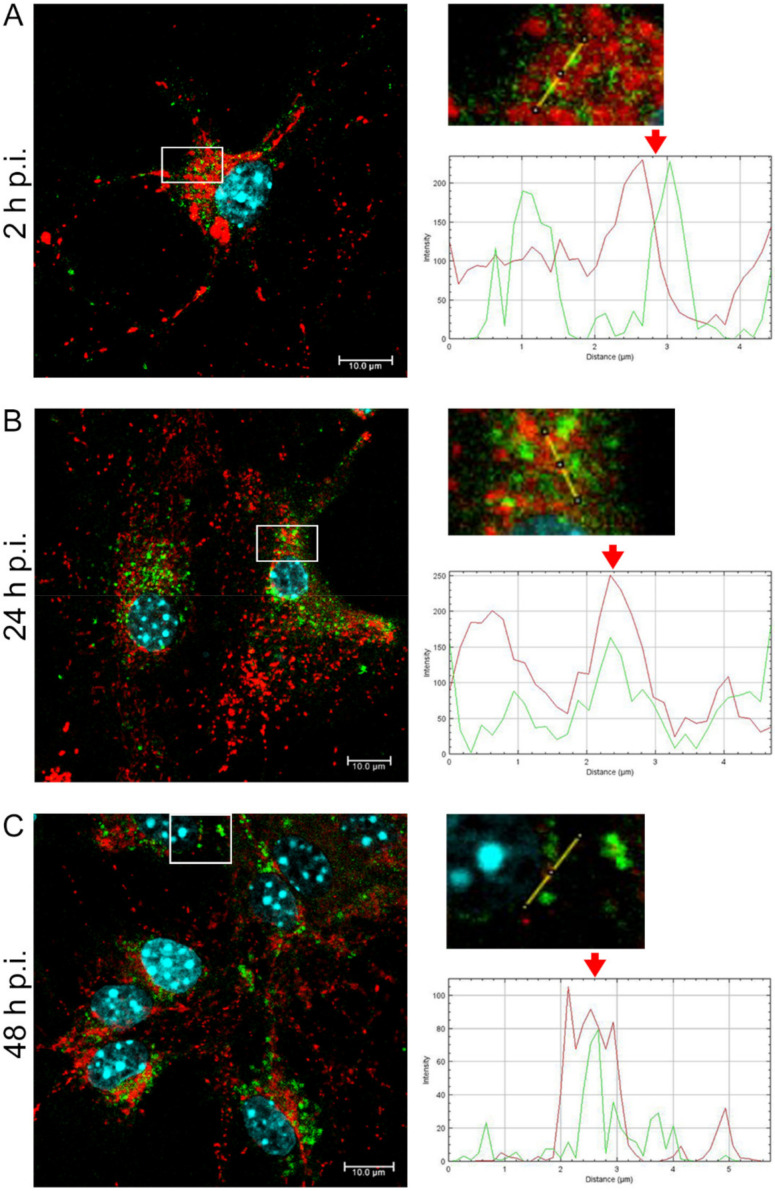
Rac-H EHV-1 infection leads to fragmented and disorganized mitochondria, with the loss of connection but the sustained branched network. Mitochondrial network morphology in neurons (**A**–**C**) at three time points (2, 24, and 48 h p.i., respectively) The line profile plots indicate the intensity distribution of green (viral antigens) and red (mitochondria) channels. Colocalization analysis was performed along yellow lines in the magnified view of ROI in the merged panel employing ImageJ software. Red arrows show the overlay of the red and green channels indicating colocalization (mitochondria—red fluorescence, viral antigens—green fluorescence, nuclei—blue fluorescence).

After infection with Rac-H EHV-1 (Figure 3), an increased mitochondrial network fission was observed at 2, 24, and 48 h p.i. We also found the occurrence of swollen mitochondria at 2 h p.i. (Figure 3A), a colocalization of viral antigens with punctate mitochondria at 24 h p.i., (Figure 3B) and a partial colocalization at 2 and 48 h p.i. (Figure 3A,C). Moreover, accumulation of viral antigens in the perinuclear space was detected at all tested time-points, and EHV-1 antigens were found in the peripheral parts of the cells at 24 and 48 h p.i. 

**Figure 4:**
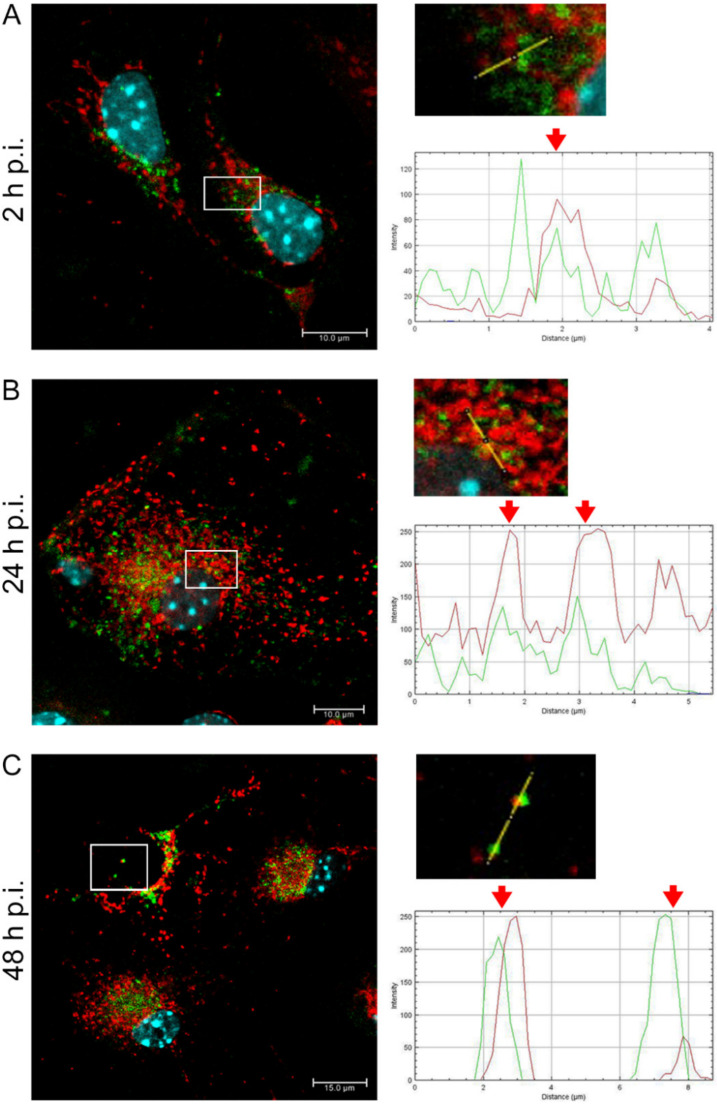
EHV-1 26 infection leads to fragmented mitochondria and increased number of single punctate mitochondria and in groups. Mitochondrial network morphology in neurons (**A**–**C**) at three time points (2, 24, and 48 h p.i., respectively). The line profile plots indicate the intensity distribution of green (viral antigens) and red (mitochondria) channels. Colocalization analysis was performed along yellow lines in the magnified view of ROI in the merged panel employing ImageJ software. Red arrows show the overlay of the red and green channels indicating colocalization (mitochondria—red fluorescence, viral antigens—green fluorescence, nuclei—blue fluorescence).

After infection with EHV-1 26 (Figure 4), we observed increased mitochondrial network fission at 24 and 48 h p.i. (Figure 4B,C), occurrence of swollen mitochondria at 2 and 24 h p.i. (Figure 4A,B) and complete colocalization of viral antigen with punctate mitochondria at 2 and 24 h p.i. (Figure 4A,B), and partial colocalization at 48 h p.i. (Figure 4C). Similarly as for other strains, viral antigens were detected in the perinuclear space at all tested time-points, while EHV-1 antigens were detected in the peripheral parts of the cells at 24 and 48 h p.i.

### 2.2. Analysis of the Mitochondrial Network

**Figure 5:**
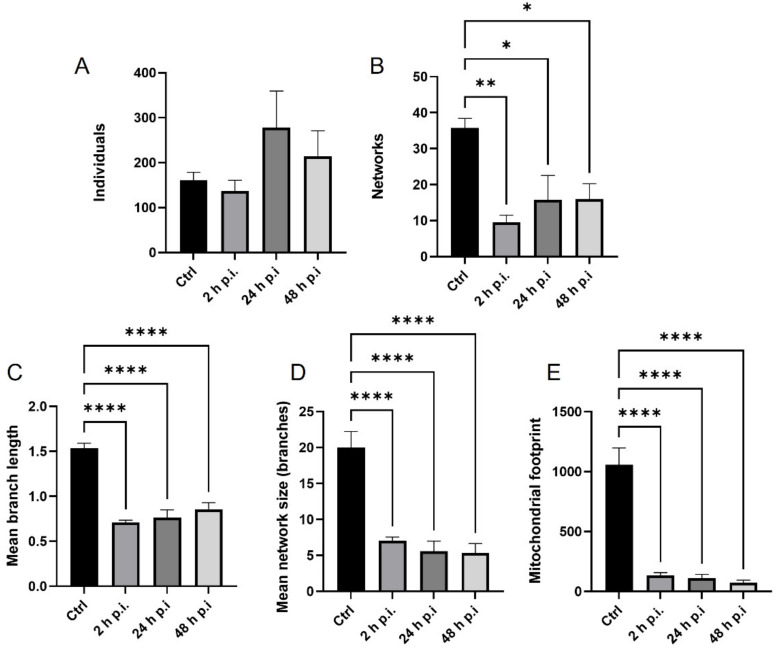
Quantitative analysis of mitochondrial morphological forms occurring post Jan-E EHV-1 infection using MiNa Single Image macro tools. The number of individuals (**A**), number of networks (**B**), mean branch length [µm] (**C**), mean network size per branches [µm] (**D**), and mitochondrial footprint [µm^2^] (**E**) were examined. The graph shows a summary statistic of infected neurons and control cells. Data from three independent experiments are presented as mean ± SEM (n = 20 cells). Turkey’s multiple comparison test *p* ≤ 0.05 *, *p* ≤ 0.01 **, and extremally significant at *p* ≤ 0.001 *** or *p* ≤ 0.0001 ****.

MiNA Single Macro Tools were used to evaluate quantitative and morphological changes in the mitochondrial network during infection. As a result of infection with the Jan-E EHV-1, neuronal cells showed an increased number of single-point mitochondria (Figure 5A). The highest number of punctate mitochondria was observed at 24 h p.i. In addition, a highly statistically significant change in the number of mitochondrial networks was detected. The most significant decrease in this number was observed as early as two hours post infection (*p* ≤ 0.001) (Figure 5B). In the case of Jan-E EHV-1 infection, highly statistically significant changes in the length of tubular mitochondria were also observed. The highest decrease in the length of 1 µm mitochondria relative to control was observed at 2 h p.i. (*p* ≤ 0.0001), (Figure 5C). There was a statistically significant decrease in the mitochondrial area after viral infection at all time points (*p* ≤ 0.0001), (Figure 5E). 

**Figure 6:**
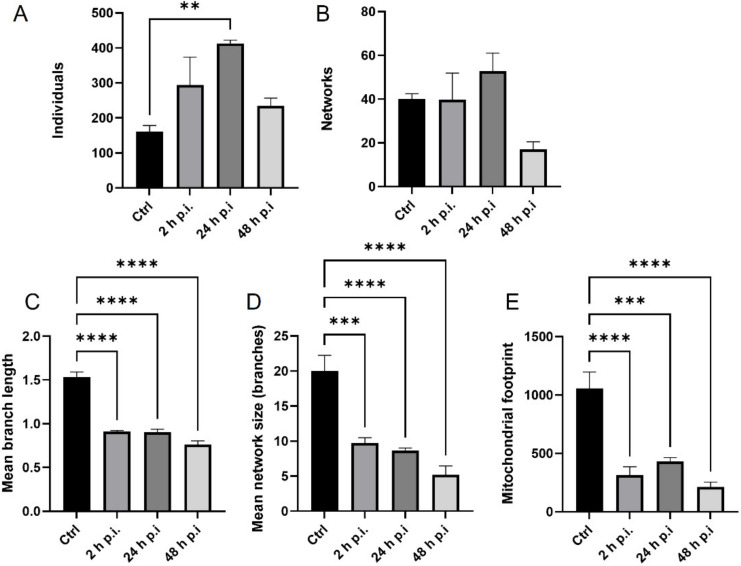
Quantitative analysis of mitochondrial morphological forms occurring post Rac-H EHV-1 infection using MiNa Single Image macro tools. The number of individuals (**A**), number of networks (**B**), mean branch length [µm] (**C**), mean network size per branches [µm] (**D**), and mitochondrial footprint [µm^2^] (**E**) were examined. The graph shows a summary statistic of infected neurons and control cells. Data from three independent experiments are presented as mean ± SEM (n = 20 cells). Turkey’s multiple comparison test *p* ≤ 0.05 *, *p* ≤ 0.01 **, and extremally significant at *p* ≤ 0.001 *** or *p* ≤ 0.0001 ****.

After infection with the Rac-H strain, a statistically significant increase in the number of punctate mitochondria was observed (*p* ≤ 0.01) (Figure 6A). Furthermore, a decrease in the number of network forms (Figure 6B) was observed only at 48 h p.i. In addition, there was a highly statistically significant decrease in the mean branch length (*p* ≤ 0.0001) (Figure 6C) mean network size (*p* ≤ 0.001 and *p* ≤ 0.0001). (Figure 6D), and mitochondrial area (*p* ≤ 0.001 and *p* ≤ 0.0001), (Figure 6E) was observed.

**Figure 7:**
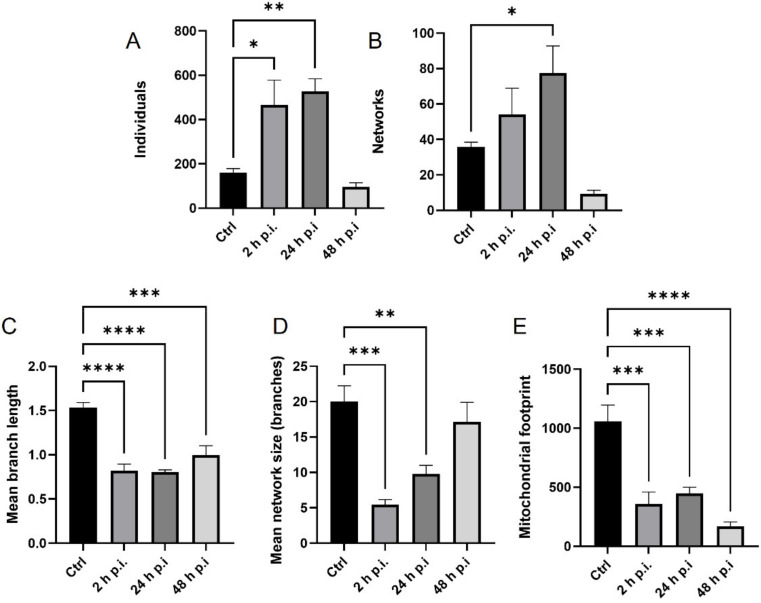
Quantitative analysis of mitochondrial morphological forms occurring post EHV-1 26 infection using MiNa Single Image macro tools. The number of individuals (**A**), number of networks (**B**), mean branch length [µm] (**C**), mean network size per branches [µm] (**D**), and mitochondrial footprint [µm^2^] (**E**) were examined. The graph shows a summary statistic of infected neurons and control cells. Data from three independent experiments’ repetition are presented as mean ± SEM (n = 20 cells). Turkey’s multiple comparison test *p* ≤ 0.05 *, *p* ≤ 0.01 **, and extremally significant at *p* ≤ 0.001 *** or *p* ≤ 0.0001 ****.

The EHV-1 26 caused an increase in the number of punctate mitochondria compared to uninfected cells (Figure 7A). A statistically significant increase occurred at 24 and 48 h p.i. (*p* ≤ 0.05; *p* ≤ 0.01). For cross-linked mitochondria, the number of mitochondria did not decrease until 48 h post infection, and this change was statistically significant compared to control cells (Figure 7B). There was a slight increase in the number of cross-linked mitochondria at 2 and 24 h p.i. A highly statistically significant decrease in the average length of tubular mitochondria was also observed (*p* ≤ 0.0001) (Figure 7C). Infection with EHV-1 26 also caused a statistically significant decrease in the average number of branches in the mitochondrial network (*p* ≤ 0.001 and *p* ≤ 0.0001) (Figure 7D). A statistically significant reduction in the area of mitochondria in the cell was evident 2 and 24 h after infection. In contrast, the area was most intensely reduced at 48 h p.i (*p* ≤ 0.0001) (Figure 7E). 

### 2.3. Assessment of Mitochondrial Mass during EHV-1 Infection

**Figure 8:**
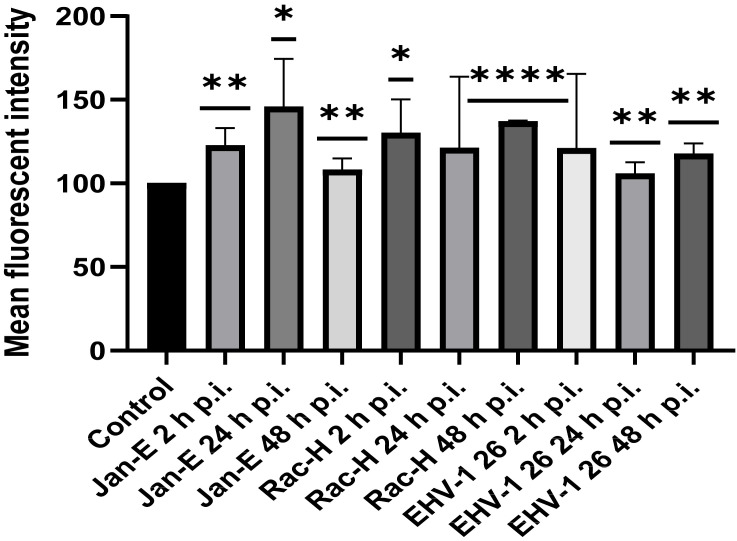
Mitochondrial mass in primary murine neurons during EHV-1 infection (2, 24, and 48 h p.i.). Mean fluorescence intensity (MFI) of MitoTracker Green TM at 2, 24, and 48 h p.i. in primary murine neurons. Data from three independent experiments are presented as mean ± SEM (n = 10,000 cells). Turkey’s multiple comparison test *p* ≤ 0.05 *, *p* ≤ 0.01 **, and extremally significant at *p* ≤ 0.001 *** or *p* ≤ 0.0001 ****.

The mitochondrial mass was detected using flow cytometric analysis of live neurons (Figure 8). After infection with the Jan-E EHV-1, significant changes in mitochondrial mass were observed. Two hours after infection, we observed an increase in mitochondrial mass in comparison to uninfected control (100%). This value was 122.51% ± 10.61 (*p* ≤ 0.01) compared to uninfected cells. After 24 h p.i., the mitochondrial mass still increased, and its value was 145.81% ± 28.65 (*p* ≤ 0.05) comparing to the control. On the other hand, after 48 h p.i., a decrease in mitochondrial mass was observed comparing to the previous measurement point. The mitochondrial mass value at this time point was 121.86% ± 6.88 (*p* ≤ 0.01). Changes in mitochondrial mass were also noted upon infection with the Rac-H. At 2 h p.i., an increase in mitochondrial mass of 130.02% ± 20.19 (*p* ≤ 0.05) was observed relative to the positive control. In contrast, at 24 h p.i., mitochondrial mass decreased relative to the previous measurement point, albeit not significantly (9%). After 48 h p.i., the mitochondrial mass increased again and reached 136.91% ± 42.64 (*p* ≤ 0.0001) compared to uninfected cells. Infection with the EHV-1 26 resulted in an increase in mitochondrial mass, where the highest increase in comparison to control was observed at 2 h p.i. and this value was 165.38% ± 0.7163 of control (*p* ≥ 0.01). After 24 h p.i., a marked decrease in mitochondrial mass was observed relative to the previous time point. This difference was 60% ± 6.69 (*p* ≤ 0.01). Moreover, at 48 h p.i., a renewed increase in mitochondrial mass was observed to 117.59% ± 6.284 (*p* ≤ 0.01) in comparison to the control (Figure 8).

### 2.4. Evaluation of the Mitophagy Process in EHV-1 Infected Neurons

**Figure 9:**
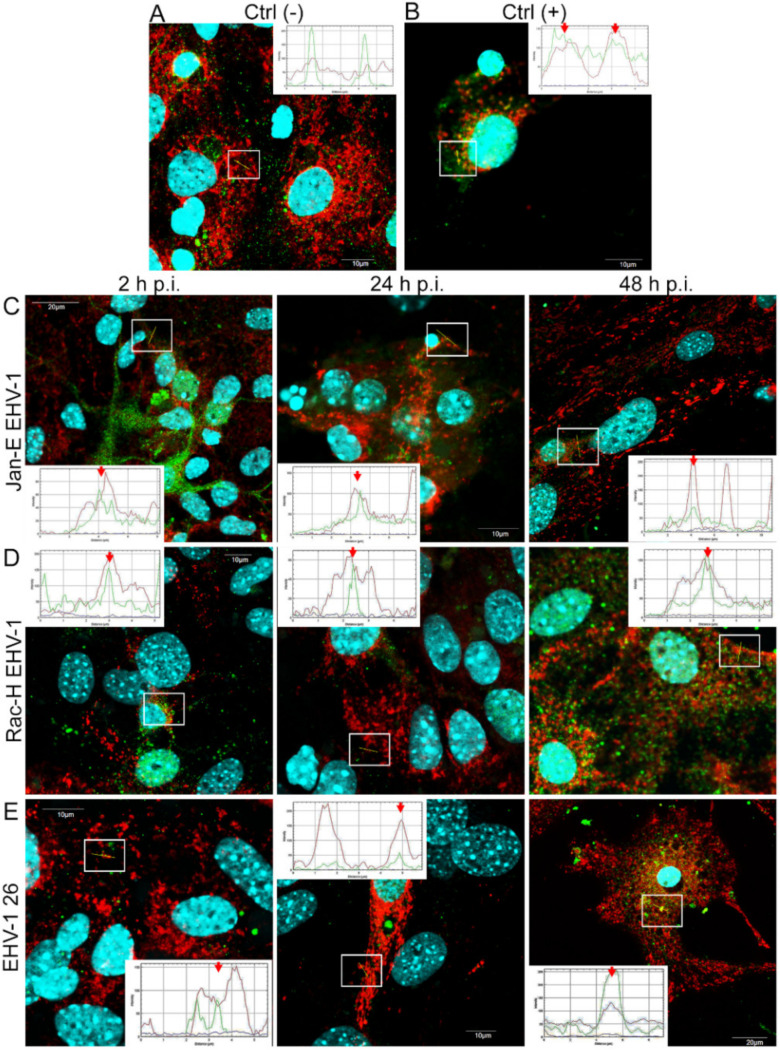
EHV-1 modulates autophagy in primary murine neurons. Immunofluorescence images of LC3B protein in uninfected neuron cells (**A**), Jan-E EHV-1 (**C**), Rac-H EHV-1 (**D**), and EHV-1 26 (**E**) infected neurons at 2, 24, and 48 h p.i. Uninfected cells pre-treated with 30 µM rapamycin for 24 h were used as a positive control (**B**). White squares indicate regions where colocalization analysis was performed employing ImageJ software. Red arrows show the overlay of the red (mitochondria) and green (LC3B) channels, indicating colocalization (mitochondria—red fluorescence, LC3B—green fluorescence, nuclei—blue fluorescence).

Upon infection with Jan-E EHV-1, we observed a progressive decrease in the amount of LC3B protein present in the form of aggregates, partially colocalizing with mitochondria at 2 and h p.i. and completely colocalizing at 48 h p.i. (Figure 9C) Infection with the Rac-H EHV-1 resulted in increased amounts of LC3B protein during infection, with a particular increase at 48 h p.i. (Figure 9D). In addition, globular forms of the protein corresponding to autophagosomes were observed. In contrast, infection with EHV-1 26 resulted in an increase in the amount of LC3B protein throughout the cell, particularly at 48 h p.i., where a spherical structure surrounding the punctate mitochondria was also observed, which may indicate the presence of selective autophagy or mitophagy (Figure 9E). Colocalization of viral antigen with LC3B protein was also confirmed by the ImageJ program analysis. Overlap of the red channel with the green channel presented as the line profile plot indicated colocalization of the LC3B protein with EHV-1 antigens (Figure 9, red arrows).

**Figure 10:**
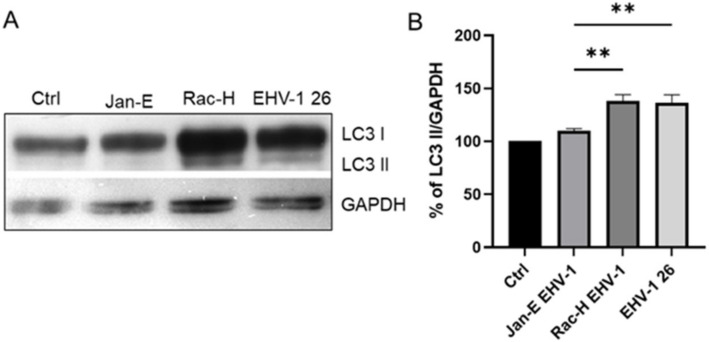
Western blot detection of LC3 I/II in EHV-1- infected neurons at 24 h p.i GAPDH represents a loading control (**A**). (**B**) Densitometric analysis of LC3II protein levels normalized to GAPDH level in mock-infected and EHV-1-infected neurons; results were statistically compared to mock-infected control. Turkey’s multiple comparison test *p* ≤ 0.01 **.

Three independent Western blot experiments showed an increased LC3 I/II protein level. The most significant increase in LC3 II protein levels was observed after infection with the Rac-H EHV-1 after 24 h p.i. Statistically significant differences in LC3 II levels were noted between the strains (Figure 10).

### 2.5. Analysis of Mitochondrial Membrane Potential during EHV-1 Infection in Neurons

**Figure 11:**
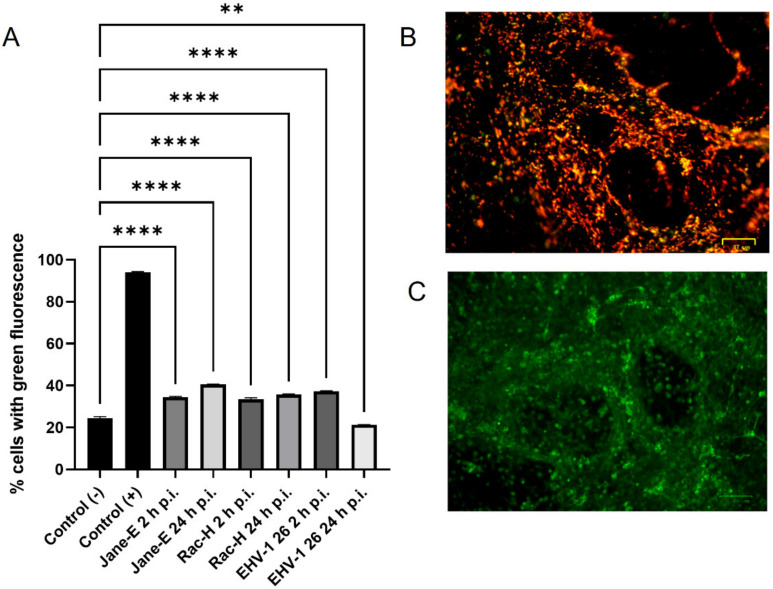
Mitochondrial membrane potential in primary murine neurons. (**A**) Percentage of cells with decreased inner mitochondrial membrane potential (green fluorescence) in primary neurons infected with Jan-E EHV-1, Rac-H EHV-1 and EHV-1 26 at different time points. (**B**) Uninfected control cells have a high mitochondrial potential (negative control), and CCCP-treated neurons ((**C**), positive control) have a low mitochondrial potential. Data from three independent experiments are presented as a mean percentage of cells with green fluorescence ± SEM are indicated on bar charts (**A**). Turkey’s multiple comparison test *p* ≤ 0.05 *, *p* ≤ 0.01 **, and extremally significant at *p* ≤ 0.001 *** or *p* ≤ 0.0001 ****.

The highest decrease in mitochondrial potential values was observed in CCCP-treated cells (positive control). EHV-1 infection led to a significant decrease in mitochondrial potential compared to control cells (Figure 11A). Upon infection with the Jan-E EHV-1 (2 h p.i.), the percentage of cells with reduced mitochondrial potential (showing green fluorescence) was 34.51 ± 0.58 (*p* ≤ 0.0001). At 24 h p.i., there was an increase in the number of cells showing a reduced inner mitochondrial membrane potential. This increase was 6% (*p* ≤ 0.0001) compared to the previous time point. A decrease in the mitochondrial inner-membrane potential was also observed during infection with the Rac-H EHV-1. This decrease was similar at both 2 and 24 h p.i. The range of cells showing green fluorescence at 2 and 24 h p.i. was 30 to 40% (*p* ≤ 0.0001). In contrast, infection with EHV-1 26 caused the greatest decrease in IMM potential at 2 h p.i. The percentage of cells showing green fluorescence was 37.3% ± 0.44 (*p* ≤ 0.0001), a statistically significant value. Interestingly, after 24 h p.i., the pool of cells showing green fluorescence returned to 21.36 % ± 0.09 (*p* ≤ 0.01) (Figure 11A).

### 2.6. Analysis of the Number of Reactive Oxygen Species during EHV-1 Infection in Neurons

**Figure 12:**
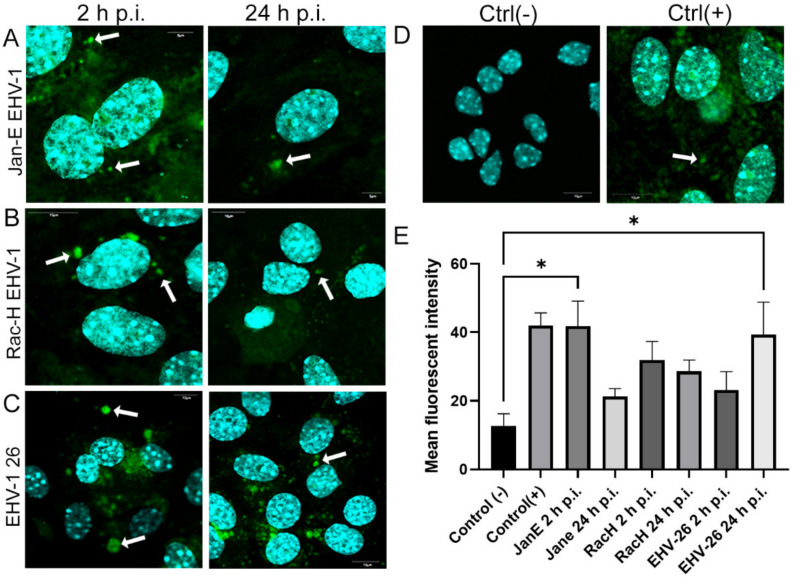
Increase in reactive oxygen species (ROS) production in primary murine neurons during Jan-E EHV-1 (**A**), Rac-H (**B**), and EHV-1 26 (**C**) infection (2 and 24 h p.i.), measured with the CellROX® Green Reagent. (**D**) Uninfected neurons were used as a negative control, while uninfected neurons treated with 1 mM H_2_O_2_ were used as a positive control. (**E**) Data from three independent experiments are presented as a mean fluorescence intensity ±SEM. Turkey’s multiple comparison test *p* ≤ 0.05 *, *p* ≤ 0.01 **, and extremally significant at *p* ≤ 0.001 *** or *p* ≤ 0.0001 ****.

Infection with all EHV-1 strains resulted in reactive oxygen species (ROS) accumulation in the cell in addition to the mitochondrial changes described previously. Reactive oxygen species were uniformly present throughout the cell at 2 and 24 h p.i. The highest amount of green fluorescence (for ROS) was observed at 2 h p.i. in neurons infected with Jan-E EHV-1 (*p* ≤ 0.05) (Figure 12E). The result was similar to the positive control, which was hydrogen peroxide-treated cells. An equally high value for ROS was recorded in neurons infected with EHV-1 26 at 24 h p.i. (*p* ≤ 0.05) (Figure 12E). For the non-neuropathogenic strains Jan-E and Rac-H, the fluorescence intensity decreased at the second measurement point, at 24 h p.i., whereas infection with the neuropathogenic strain n was characterized by an increase in the amount of ROS at both tested times, with the highest intensity observed at 24 h p.i. (Figure 12E). 

### 2.7. Analysis of Mitochondrial Gene Expression during EHV-1 Infection in Neurons

**Figure 13:**
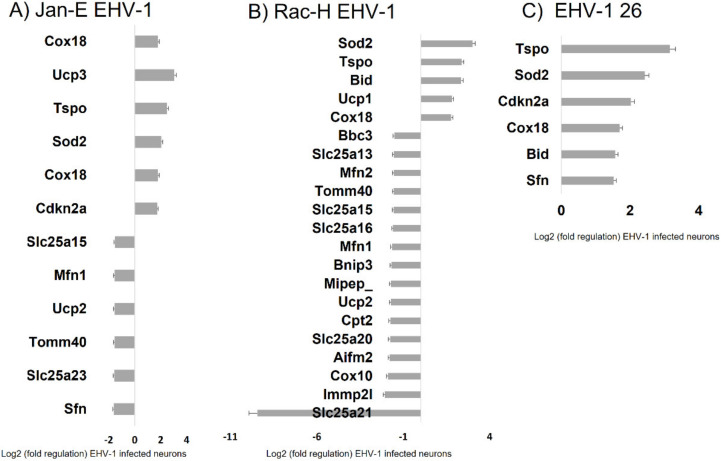
Expression of mitochondrial genes in primary murine neurons infected with Jan-E (**A**), Rac-H (**B**), EHV-1 26 (**C**). Quantitative data are expressed as mean ± standard deviation (SD) from three independent biological replicates. Real-time PCR was performed using 96-well Mitochondria RT^2^ profiler PCR array plates (Qiagen).

Proteins associated with the fission and fusion of mitochondria are essential for function of these organelles. At 24 h p.i. with Jan-E EHV-1, a decrease in the expression of the mitochondrial fusion-related gene Mitofusin-2 (Mfn2) was observed (Figure 13). In contrast, there was an almost two-fold increase in the expression of the Cytochrome C Oxidase Assembly Factor COX18 gene (Cox18). The protein encoded by Cox18 is responsible for protein integration into the mitochondrial membrane and cytochrome oxidase activity. Moreover, infection with Jan-E EHV-1 was followed by a more than two-fold increase in expression of the gene encoding Translocator Protein (Tspo), which plays an important role in the transport of cholesterol to mitochondria and is involved in their metabolism. A three-fold increase in Uncoupling Protein 3 (Ucp3) gene expression and a decrease in Uncoupling Protein 2 (Ucp2) gene expression were also observed. These genes encode proteins of the same name responsible for the so-called proton leakage across the inner mitochondrial membrane; consequently, the lack of a conserved gradient results in a decrease in the amount of ATP formed. Moreover, these proteins are involved in the transport of anions from the inner to the outer mitochondrial membrane and the return transfer of protons from the outer to the inner mitochondrial membrane. They also affect the reduction in the mitochondrial membrane potential. Infection with the Jan-E EHV-1 also caused a decrease in the expression of genes encoding proteins related to small molecule transport. This affected the Solute Carrier Family 25, Member 21 (Slc25a21), Solute Carrier Family 25, Member 22 (Slc25a22), Solute Carrier Family 25, Member 23 (Slc25a23), Solute Carrier Family 25, and Member 15 (Slca25a15) genes. The expression levels of genes related to import and mitochondrial targeting proteins were also altered. After infection with the Jan-E EHV-1, there was a decrease in the expression level of the Stratifin (Sfn) gene. The encoded protein is responsible for cell cycle regulation. It also participates in the regulation of translation by binding to translation factors. In addition, it prevents errors in DNA during mitotic division. 

For proteins associated with membrane translocation, we found a decrease in the expression of the two genes Translocase Of Outer Membrane 40 (Tomm40) and Inner Mitochondrial Membrane Peptidase 2 Like (Immp2l), described previously. At 24 h p.i. with the Rac-H EHV-1, changes in the expression of genes related to the fusion and fission process were observed. There was a decrease in Cox10, Mitofuzin-1 (Mfn1), and Mfn2 gene expression. In contrast, the level of the Cox18 gene almost doubled. The Cytochrome C Oxidase Assembly Factor Heme A: Farnesyltransferase COX10 (Cox 10) encodes heme A: farnesyltransferase, which is not a structural subunit of cytochrome oxidase but is required for the expression of functional COX. In addition, there were changes in the expression of genes related to mitochondrial membrane potential and polarity. Uncoupling Protein 1 (Ucp1) and Tspo genes showed increased expression relative to controls. 

In contrast, Ucp2 and Bnip3 genes had a 1.5-fold decrease in expression. The BCL2 Interacting Protein 3 (Bnip3) gene encodes a protein that acts as an anti-apoptotic factor. By interacting with Spermatogenesis-Associated 18 Homolog (SPATA18/MIEAP), Bnip3, and BNIP3L/NIX proteins found on OMM, it regulates pore opening in the mitochondrial membrane to mediate translocation of lysosomal proteins from the cytoplasm to the mitochondrial matrix. A decrease in the expression of genes related to small molecule transport was also observed. The decrease involved the genes: Slc25a21, Solute Carrier Family 25, Member 20 (Slc25a20), Solute Carrier Family 25, Member 13 (Slc25a13), Slc25a15, Solute Carrier Family 25, and Member 16 (Slca25a16). The greatest decrease in expression was observed for the Slc25a21 gene (9-fold decrease). The expression of genes related to mitochondrial transport also changed after infection with the Rac-H EHV-1. The Inner Mitochondrial Membrane Peptidase Subunit 2 (Immp2l) gene showed a two-fold decrease in expression. This gene encodes a protein that directs peptides from the mitochondrial matrix through the IMM. In addition, there was also an almost two-fold decrease in the level of the Mitochondrial Intermediate Peptidase (Mipep) gene. It encodes a protein that catalyzes the transport of nuclear proteins into the mitochondrion. It is also involved in the maturation of proteins associated with oxidative phosphorylation. In the group of genes related to mitochondrial transport, an increase in expression was observed only for the Tspo gene. 

The expression of genes related to apoptosis also changed. A decrease in expression occurred for Apoptosis-Inducing Factor Mitochondria-Associated 2 (Aifm2), Bnip3, and Bcl2-Binding Component 3 (Bbc3) genes, while an increase was observed for Superoxide Dismutase 2 (Sod2). The Aifm2 gene encodes a flavoprotein oxidoreductase that binds single-stranded DNA and can cause apoptosis due to viral infection. In turn, the product of the Bbc3 gene affects OMM permeability and binds to other Bcl2 family proteins to induce apoptosis and caspase activation. When infected with EHV-1 26, no large changes were observed in the expression levels of genes related to mitochondrial biogenesis and function. Only an increase in the expression of six genes was observed at 24 h p.i.: Sfn, Bid, COX18, Cyclin-Dependent Kinase Inhibitor 2A (Cdkn2a), Sod2, Tspo (Figure 13).

## 3. Discussion

EHV-1 is the etiological agent causing, in addition to mild inflammation of the lower respiratory tract, a disease syndrome named equine herpesvirus myeloencephalopathy (EHM). Infected horses exhibit a range of neurological signs, ending with a high mortality rate. The exact pathogenesis of EHV-1-induced CNS infections is not fully understood, so it seems important to study how EHV-1 influences nerve cells and endothelial cells of blood vessels that form the blood–brain barrier [16,17,18,19]. Here, we showed that after infection with all EHV-1 strains, changes in the mitochondrial network morphology occurred and were manifested mainly by its fission. The described changes appeared in the initial period of infection, i.e., at 2 h p.i. It can be assumed that they are connected with the migration of the virus to the cell nucleus to perform replication. The virus migrating to the nucleus uses elements of the cell cytoskeleton, which has been confirmed in our previous studies [14,20]. Indeed, there is a possibility of inhibition of mitochondrial distribution, as it is supposed that mitochondria can "compete" with virions for space in the cytoskeleton. Murata et al. have shown that in Vero cells, mitochondria are recruited to the site of HHV-1 replication and morphogenesis. They have also demonstrated that mitochondria migrate to the perinuclear area where HHV-1 tegument was present. It is possible that mitochondria, as energy centers of the cell, provide the energy necessary for the replication of the virus. It has also been shown that the mitochondrial potential is stable up to 6 h post infection but decreases during the late phase of infection [21]. Our studies show that EHV-1 infection causes an increase in mitochondrial punctate forms. Furthermore, we observed partial or complete colocalization of viral antigen with mitochondria. In our previous studies in a model of HHV-1 and HHV-2 infection, we obtained similar results. In HHV-1- and HHV-2-infected neurons, we observed increased fusion of the mitochondrial network. Furthermore, the translocation of the cytoplasmic protein Drp1 from the cytoplasm to the mitochondrial membrane was observed [21,22]. Studies on other herpesviruses also indicate that mitochondrial size changes during infection. Kramer et al. (2012) showed a decrease in mitochondrial size during SuHV-1 (SuHV-1, Aujeszky’s disease virus, ADV) infection [23]. In addition, studies by Saffran et al., 2007 showed that it is possible to eliminate mtDNA and replace it with viral genetic material during the productive cycle. These studies shed new light on the role of mitochondria during viral infection. Damage to mtDNA accompanies many neurodegenerative diseases, so it is critical to determine the factors that influence mtDNA destruction during infection [11]. Furthermore, swollen-like mitochondria, also known as megamitochondria, were observed during EHV-1 infection. This morphological type is associated with changes in permeability of ion channels, especially the so-called calcium megacanals [24]. It has been experimentally demonstrated that both increased calcium ion concentration and the occurrence of free radicals cause permeability disruption of mitochondrial membranes, thus causing a change in their shape [25]. 

After a complete replicative cycle of EHV-1 lasting about 18 h, mitochondria appeared in a punctate form. They continued to occur near the cell nucleus, probably serving as energy donors to transport the virus toward the cell membrane in the process of exocytosis. Migration and movement of cytoskeletal elements and assembly of progeny virions are processes that require energy supply [15]. We observed perinuclear localization of mitochondria, which strongly suggest their involvement during EHV-1 virus assembly, occurring in the cell nucleus. This study remains consistent with other reports of perinuclear localization of mitochondria during infection. Hepatitis B virus (HBV), for example, produces the HBx protein that directs mitochondria toward the nucleus, even forming aggregates of these organelles [26]. The morphological change in the mitochondrial network is often associated with its current physiological state. Previous studies reported that excessive mitochondrial network fission is associated with apoptosis [27,28]. Different results were obtained by [22]. and Chodkowski et al, 2017, who showed that despite the fission of the mitochondrial network, the functioning of these organelles was preserved; moreover, during infection, some parameters such as mitochondrial potential were normalized compared to control cells, which does not indicate apoptosis. Therefore, to fully assess the functionality of mitochondria during EHV-1 infection, several indicators of the functioning of these organelles should be taken into account. In this study, we decided to investigate not only the morphology of the mitochondrial network but also such parameters as mitochondrial mass, ROS production, the occurrence of mitophagy, and changes in the level of mitochondrial gene expression during infection. 

In this study, we analyzed the production of ROS during infection. Upon infection with non-neuropathogenic Jan-E and Rac-H EHV-1, the highest increase in ROS occurred at 2 h p.i., followed by a decrease observed at 24 h p.i. Our team previously showed that the Jan-E EHV-1 and Rac-H EHV-1 induced apoptosis only in a small population of cells [14]. Different results were obtained upon infection with the EHV-1 26, where a higher amount of ROS was present at 24 h p.i. As indicated by our previous studies, infection with EHV-1 26 induces hyperphosphorylation of Tau protein, considered as an indicator of neurodegeneration. Therefore, high levels of ROS may be related with these processes [29]. The amount of ROS seems to be an important indicator for the proper functioning of mitochondria and the whole cell. Similar results were obtained for HHV-1 and HHV-2 infections, where also the amount of ROS was highest at the beginning of the infection [22]. In contrast, researchers studying viruses whose replication cycle occurs in the cytoplasm obtained different results. For ectromelia virus (ECTV), a gradual increase in the amount of ROS was observed during infection, where it reached its highest value only at the end of the infection [3]. Neurons are characterized by a much broader spectrum of mechanisms protecting against oxidative stress than other cells. It has been shown that ROS can affect axonal growth in cell lines such as PC12 and SH-SY5Y. Therefore, the infected cells where large amounts of ROS are produced may have a different morphology and a different distribution of the mitochondrial network [30].

Cytometric analysis of the mitochondrial inner-membrane potential demonstrated a decrease in IMM potential after infection. The decrease was low for Jan-E and Rac-H EHV-1 infection at both 2 and 24 h p.i. In contrast, different results were obtained for the neuropathogenic strain, where the decrease was more pronounced at the initial stage of infection and the IMM potential normalized at the final stage, relative to the negative control. The results obtained upon infection with the EHV-1 26 are consistent with HHV-1 and HHV-2 infection, where a higher decrease in potential was also observed very early during infection. For other viruses, such as ECTV, a gradual decrease in potential was observed with the progression of infection [3,22]. Furthermore, several authors described various viral proteins localizing to the IMM or OMM and disrupting the electrochemical gradient necessary for ATP synthesis [31]. For example, HIV R protein (Vpr protein) localizes to mitochondria and binds to ANT (adenine nucleotide translocase), causing a decrease in IMM potential. The influenza virus type A protein PB1-F2, in turn, interacts with anion channels, thereby causing a decrease in the potential and activation of proapoptotic proteins [3,22,31,32,33].

For all three EHV-1 strains there was an increase in expression of three genes: *Sod2, Tspo*, and *Cox18*. The *Sod2* (superoxide dismutase 2) protein is responsible for reducing the number of reactive oxygen species and converting to hydrogen peroxide [34]. The increase in expression of this gene is in line with the demonstrated increase in ROS after EHV-1 infection. An increase in the expression of the *Cox18* gene related to cytochrome c oxidase was also observed. The product of this gene enables the translocation of the oxidase subunit across the inner membrane. Therefore, it is important for the proper conduct of electron transport along the respiratory chain [35]. Another gene that was down-regulated during infection with all EHV-1 strains was *Tspo*, responsible for mitochondrial transport. Experimentally, the Tspo protein has been shown to play a key role in the assembly of the HIV envelope, specifically its Env glycoprotein component. A more than 64-fold increase in the expression of this gene was observed in HIV-infected cells. Interestingly, when the *Tspo* gene is turned off, the resulting glycoprotein is rapidly degraded [36]. EHV-1 antigens also localized near mitochondria and partially colocalized with them, which may also indicate that mitochondria are involved in the production of viral envelope elements. The Tspo protein is also considered a biomarker for neurodegenerative diseases such as Alzheimer’s disease. For EHV-1 infection, the increase in expression of this gene was highest in the neuropathogenic strain EHV-1 26, which also makes it a good marker for neuroinfections with EHV-1 etiology.

In summary, we demonstrated that EHV-1 infection of neuronal cells leads to specific morphological and functional modifications at the early stage of infection, followed by colocalization of mitochondria with EHV-1 in the perinuclear area. The results indicate that mitochondria play an important role during EHV-1 replication both at the early stage and later during infection. 

## 4. Materials and Methods

### 4.1. Neuronal Culture

Balb/c (H-2d) mice susceptible to EHV-1 infection were used to establish primary culture of murine neurons, according to the procedure described previously [6,12]. Neurons were cultured in B-27 neuron plating medium (neurobasal medium, B27 supplement, 200 mM glutamine, and 10 mM glutamate) supplemented with antibiotics (penicillin and streptomycin), 10% fetal bovine serum (FBS) and 5% horse serum (HS) (Gibco, Thermo Fisher Scientific, Waltham, MA, USA). The cells were maintained 37 °C with 5% CO_2_. At day 3 post plating, cells were treated with 10 μM cytosine β-D-arabinofuranoside (Sigma-Aldrich, St. Louis, MO, USA) for 24 h to eliminate non-neural cells. Next, the medium was removed and replaced with neuron feeding medium (B-27 neuron plating medium without glutamate). Prior to treatments, the cells were cultured for 6 days at 37 °C with 5% CO_2_.

### 4.2. Viruses and Cells Infection

Three different strains of EHV-1 were used to infect neuronal cultures: non-neuropathogenic, reference Rac-H (149th passage in ED cells) and non-neuropathogenic Jan-E (12th passage in ED cells) strains isolated from the aborted fetus (neuropathogenicity confirmed by a PCR-RFLP neuropathogenic/non-neuropathogenic discrimination test) [15], and a neuropathogenic EHV-1 strain (EHV-1 26, 15th passage in Vero cells) isolated from an aborted fetus in Hungary in 2004 (neuropathogenicity confirmed by PriProET technique) [37]. The viruses were propagated in equine dermal (ED) and Vero cell cultures, as described previously [6,12]. Neurons were infected with Jan-E, Rac-H, or EHV-1 26 EHV-1 strains at MOI (multiplicity of infection) of 1.0 for 60 min at 37 °C. Neurons were collected at 2, 24, or 48 h p.i.

### 4.3. Immunofluorescent Labeling and Confocal Microscopy Analysis

Primary murine neurons seeded on glass coverslips (coated with poly-D-lysine and laminin) were infected with Jan-E, Rac-H, or EHV-1 26. At 2, 24, and 48 h post infection (h p.i.), cells were incubated for 30 min at 37 °C with 100 nM MitoRed (Sigma-Aldrich) to visualize the mitochondrial network morphology and distribution. To detect the colocalization of EHV-1 antigens and LC3B, cells were fixed with 3.7% PFA in PBS for 20 min. Fixed cells were permeabilized with 0.5% Triton X-100 (Sigma-Aldrich) in PBS (15 min) and blocked with 1.5% bovine serum albumin (BSA, Sigma-Aldrich) in 0.1% Triton X-100- PBS solution (30 min) to prevent nonspecific binding. The presence of LC3B was detected by using anti-LC3B polyclonal antibody (dilution 1:500; Thermo Fisher Scientific, Waltham, MA, USA) and Alexa Fluor 488 goat anti-rabbit (dilution 1:250; Thermo Fisher Scientific, Waltham, MA, USA). The presence of viral antigen was detected by direct immunofluorescence, using polyclonal rabbit antiserum EHV-1/ERV conjugated to FITC (Veterinary Medical Research & Development, Inc, Pullman, WA, USA). Cell nuclei were visualized with Hoechst 33258 (1 μg/mL), according to the manufacturer’s recommendations. Slides were mounted in ProLong Gold Antifade Reagent (Thermo Fisher Scientific, Waltham, MA, USA). Non-infected neurons served as negative control. Confocal images were acquired using a Fluoview FV10i laser scanning confocal microscope (Olympus Poland Sp. z o.o, Wrocław, Poland) with a 60× water immersion lens. Microscopic analysis was performed using FV10i software (Olympus, Wrocław, Poland), *Fiji* ImageJ version (NIH Image, version 1.53a, USA), and Adobe Photoshop CS6 software (Adobe Systems Incorporated, San Jose, CA, USA).

### 4.4. Flow Cytometry Analysis

Flow cytometry was used to measure mitochondrial mass in EHV-1 infected neurons with MitoTracker Green FM (490/516 nm [Em/Ex]). Neurons (10^6^ cells/mL) at 2, 24, and 48 h p.i. were stained with MitoTracker Green FM (200 nM; Thermo Fisher Scientific, Waltham, MA, USA) for 10 min in 37 °C, according to the manufacturer’s protocols. It is a non-fluorescent dye in aqueous solutions, but becomes fluorescent once it accumulates in the lipid environment of mitochondria, regardless of membrane potential. Samples were analyzed by BD LSR Fortessa cytometer (BD Biosciences, Franklin Lakes, NJ, USA). Non-infected neurons stained with MitoTracker Green FM were used as a positive control and non-infected neurons unstained with MitoTracer Green FM were used as a negative control.

### 4.5. Image Cytometry Analysis 

To evaluate mitochondrial membrane potential and neuronal vitality after EHV-1 infection, we used NucleoCounter NC-3000 image cytometer (ChemoMetec, Lillerød, Denmark). The cultured neurons were stained with JC-1 (cationic dye 5,5,6,6-tetrachloro-1,1,3,3-tetraethylbenzimidazolcarbocyanine iodide; ChemoMetec A/S, Lillerød, Denmark) according to the manufacturer’s protocol. First, suspended cells (non-infected and infected) were diluted with PBS to a final concentration of 1.5 × 10^6^ cells/mL. The samples were then incubated with 12.5 μL of 200 mg/mL JC-1 for 10 min at 37 °C. Following incubation, cells were washed twice and at the end of the second washing, samples were resuspended in 250 μL of 1 mg/mL 4′,6- diamidino-2-phenylindole in PBS. Next, the samples were analyzed with the NucleoCounter NC-3000, according to the manufacturer’s protocols. The results were analyzed using the NucleoView NC-3000 software (www.chemometec.com, online access on 3 July 2018). A positive control for mitochondrial potential analysis was prepared by adding CCCP (carbonyl cyanide m-chlorophenylhydrazone; 5 μl/ml cell culture medium). Non-infected neurons served as a negative control.

### 4.6. Measurement of Reactive Oxygen Species Level

Reactive oxygen species (ROS) levels were measured with the CellROX^®^ Green Reagent (Thermo Fisher Scientific, Waltham, MA, USA), a fluorogenic probe for measuring oxidative stress in live cells (Ex/Em~485/520 nm), according to the producer’s protocol. A total of 5 μM CellROX^®^ Green Reagent and Hoechst 33342 were added to the complete media of both control and EHV-1-infected neurons and incubated at 37 °C for 30 min. After washing with PBS, cells were analyzed using a confocal microscope (Fluoview FV10i, Olympus, Wrocław, Poland). Images were captured and converted to 24-bit tiff files for visualization with FV10i software (Olympus, Wrocław, Poland). Uninfected neurons treated with 1 mM H_2_O_2_ were used as a positive control.

### 4.7. RNA Isolation and Quantitative PCR

RNA was isolated from neuron cells using the Qiagen RNAeasy Mini Kit (Qiagen, Germantown, MD, USA), as recommended by the manufacturer. The RNA concentration was measured using the Take-3 system on an Epoch BioTek spectrophotometer and quantified using Gen5 software (BioTek Instruments, Inc, Winooski, VT, USA). Real-time PCR was performed using 96-well Mitochondria RT^2^ profiler PCR array plates (Qiagen Germantown MD, USA) and ABI 7500 thermocycler (Life Technologies, Carlsbad, CA, USA) at 95 °C for 10 min, 40 cycles of 95 °C for 15 sec, and 60 °C for 1 min. Expression values were collected using the SDS Software system (Applied Biosystems). Assays were performed in three independent replicates. Gene expression was normalized to the expression values of reference genes (*GAPDH, b2m*, and *Hsp90ab1*) and evaluated against a negative control, which were uninfected cells. Each PCR array plate contained lyophilized primers for 84 genes associated with the mitochondrial functions into six categories: mitochondrial membrane potential and polarity, mitochondrial transport, transport of small molecules, targeting of proteins to mitochondria, import proteins, outer-membrane translocation, inner-membrane translocation, fusion and fission, mitochondrial localization, and apoptosis.

### 4.8. Analysis of Mitochondrial Network

MiNa Single Image macro tools were used to analyze changes in the mitochondrial network. This specific bioinformatic method allows the determination of the number of point mitochondria (individuals), cross-linked mitochondria (network), mean branch length [µm], mean network size per branches [µm], and mitochondrial area in the cell (mitochondrial footprint [µm^2^]). A protocol according to Valente et al. 2017 was used [38]. Confocal microscopy images of 20 cells per each experiment were used for analysis. 

### 4.9. Western Blot Analysis

For Western blotting, cells were harvested 24 h post infection using N-PER Neuronal protein extraction reagent (Thermo Fisher Scientific, Waltham, MA, USA). The total protein content of each cell sample was measured using the BCA protein assay kit (Thermo Fisher Scientific, Waltham, MA, USA). Following SDS polyacrylamide gel electrophoresis and electroblotting to PVDF membrane, LC3 I/II proteins were detected using polyclonal anti LC3 antibody (Thermo Fisher Scientific, Waltham, MA, USA). After several washes in 0.1% Tris-buffered saline (TBS)-Tween 20, membranes were incubated with HRP-conjugated secondary antibodies for 1 h at RT and developed using enhanced chemiluminescence (Clarity Western ECL substrate; Bio-Rad, Hercules, CA, USA). The protein bands were visualized using the ChemiDoc™ MP Imaging system (Bio-Rad, Hercules, CA, USA). Glyceraldehyde 3-phosphate dehydrogenase (GAPDH) was used as a loading control and for protein normalization during densitometry measurements. For densitometry analysis, ImageLab 6.0.1 (BioRad Hercules, CA, USA) software was used. 

### 4.10. Statistical Evaluation 

The results were statistically evaluated by one-way analysis of variation (ANOVA) followed by a Turkey’s multiple comparison test with GraphPad Prism™ version 9 software (GraphPad Software Inc., San Diego, CA, USA). Statistical differences were interpreted as significant at *p* ≤ 0.05 *, highly significant at *p* ≤ 0.01 **, and extremally significant at *p* ≤ 0.001 *** or *p* ≤ 0.0001 ****.

## 5. Conclusions

Research on mitochondria is highly relevant to virology. Data concerning changes in the structure and distribution of the mitochondrial network provide us with information on the role of these organelles in the course of viral infection. However, pinpointing a specific role for these organelles is extremely difficult, primarily because of their remarkable dynamics. They are among the few cellular organelles that are characterized by frequent changes in both morphological and internal structure. The balance between these organelles is observed depending on the current state of the cell. It is also worth noting that they are among the “more sensitive” organelles, so by analyzing them experimentally, results masking the reality can be obtained.

EHV-1 continues to play an essential role as an equine pathogen, especially the neurological form, whose mechanism of pathology is not fully understood. The work presented here enriches the current state of knowledge on the interaction of EHV-1 and mitochondria in the nerve cell, which may partially explain the molecular mechanism of virus-induced neuronal damage and degradation.

## Data Availability

Not applicable.
